# Effects of transcranial magnetic stimulation on cerebellar ataxia: A systematic review and meta-analysis

**DOI:** 10.3389/fneur.2023.1049813

**Published:** 2023-01-27

**Authors:** Ying Wang, Di Zhang, Ju Wang, Jiang Ma, Li Lu, Song Jin

**Affiliations:** ^1^School of Medical and Life Sciences, Chengdu University of Traditional Chinese Medicine, Chengdu, Sichuan, China; ^2^Department of Rehabilitation, Hospital of Chengdu University of Traditional Chinese Medicine, Chengdu, Sichuan, China; ^3^Department of Rehabilitation, Traditional Chinese Medicine Hospital of Qingyang District, Chengdu, Sichuan, China; ^4^Department of Rehabilitation, Southwest Hospital, Third Military Medical University (Army Medical University), Chongqing, China

**Keywords:** hereditary ataxias, spinocerebellar ataxias, transcranial magnetic stimulation, systematic review, treatment, meta-analysis

## Abstract

**Objective:**

To determine the effectiveness of transcranial magnetic stimulation in improving cerebellar ataxia.

**Data sources:**

PubMed, EMBASE, the Cochrane Library, Springer, Science Direct, the China National Knowledge Infrastructure (CNKI) and the China Science and Technology Journal Database (VIP) were searched until 2022.

**Review methods:**

Trials with transcranial magnetic stimulation on the effects on cerebellar ataxia were included, and the effect size was evaluated using the standardized mean difference (SMD) or mean difference (MD) and a 95% confidence interval (CI).

**Results:**

Eight studies comprising 272 participants, published between 2014 and 2022, were included. The results revealed that the effect of TMS on patients with cerebellar ataxia as assessed by the International Cooperative Ataxia Rating Scale (ICRAS), the Scale for the Assessment and Rating of Ataxia (SARA), the Berg Balance Scale (BBS), and the Timed Up and Go (TUG) test was statistically significant (*P* < 0.01) with low heterogeneity among the studies (I^2^ = 4, 27, 0, and 0% respectively).

**Conclusion:**

The effects of transcranial magnetic stimulation in improving cerebellar ataxia in the affected patients are significant. TMS targeting the cerebellar structures can induce changes in the excitability of the cerebellar-thalamus-cortical pathways; thus, it is necessary to carry out large-scale research with good design and high quality in the future.

## 1. Introduction

Cerebellar ataxia is a heterogeneous group of acquired and hereditary disorders (1). Its clinical manifestations include balance and coordination disturbances, dysarthria, oculomotor deficits, dysmetria, and kinetic tremor (2). The hereditary and sporadic forms of neurodegenerative ataxia occur more frequently in adulthood (3), and the estimated prevalence is between 1 and 3 per 100,000 people (4). Balance disorders and impaired lower limb movement caused by ataxia can lead to mobility restrictions and affect the activities of daily living. Therefore, it is essential to recover from the symptoms of cerebellar ataxia. Currently, most ataxias lack effective evidence-based treatments, although many therapeutic approaches have been attempted in recent years (5). Transcranial magnetic stimulation (TMS) is an indispensable neuroelectric and physiological method for diagnosing and evaluating many neurological diseases (6). TMS therapy can be considered an effective treatment for this group of disorders (5).

Transcranial magnetic stimulation enables the non-invasive modulation of cortical excitability by involving the cerebellar-thalamus-cortical pathway (1, 7, 8). Several recent studies demonstrated that cerebellar TMS can facilitate cortical motor activation through the modulation of Purkinje cell excitability (9–11), leading to increased inhibition of the cerebello-dentato-thalamo-cortical facilitatory connection and finally resulting in the observed inhibition of M1 (8, 12, 13). The repetitive magnetic stimulation causes the late phase of neural plasticity, stimulating gene expression and increasing protein synthesis (14). Therefore, the cerebellum, as a subcortical structure, is involved in the plasticity mechanisms of motor learning (15).

Therefore, modulating plasticity through TMS is a potential method for treating cerebellar ataxia. There are many published studies on the application of TMS for the improvement of ataxia symptoms. Further, as the TMS parameters have been optimized and the patient selection criteria have improved, an updated meta-analysis is necessary to reassess the overall impact of the TMS on ataxia symptom recovery.

## 2. Methods and analysis

### 2.1. Search strategy

We searched PubMed, Embase, the Cochrane Library, Springer, Science Direct, the China National Knowledge Infrastructure (CNKI), and the Chinese Science and Technology Periodical Database (VIP). The search terms were “cerebellar ataxias,” “transcranial magnetic stimulation”/“TMS,” “systematic review,” and “treatment,” and the bibliography lists of selected papers were checked manually as follows: ((((((Transcranial Magnetic Stimulation^*^ [Title/Abstract]) OR (Magnetic Stimulation^*^, Transcranial [Title/Abstract])) OR (Stimulation^*^, Transcranial Magnetic [Title/Abstract])) OR (TMS[Title/Abstract])) OR (rTMS [Title/Abstract])) AND (cerebellar ataxia [Title/Abstract]).

### 2.2. Study selection

We selected relevant articles for the review using the eligibility criteria based on the Population, Intervention, Comparison, Outcome, and Study (PICOS) framework as follows: patients (1) aged ≥18 years; (2) with cerebellar ataxia based on the clinical history and neurological examination; (3) who received TMS as the type of intervention; and (4) who presented in randomized controlled trials.

### 2.3. Data collection and extraction

Two reviewers independently selected the clinical trials that complied with the inclusion criteria. The screening process eliminated irrelevant and duplicated data. We extracted the following data from the eligible sources: study information (number of participants, intervention), participant characteristics (age and duration), intervention protocol (TMS, frequency, and additional interventions), and outcomes. We contacted the corresponding authors when the complete literature was unavailable or the relevant data were incomplete. Disagreements between the reviewers were resolved through discussion and reaching a consensus.

### 2.4. Quality assessment

Two reviewers assessed the risk of bias in the included trials using the Cochrane Collaboration risk assessment tool. We evaluated the risk of bias (low, unclear, or high) in seven areas, including random sequence generation, allocation concealment, blinding of participants and personnel, blinding of the outcome, assessment, incomplete outcome data, selective reporting, and other sources of bias. The methodological quality was assessed using an improved Jada scale (0–3, low rate; 4–7, high quality) (16). Any disagreements were resolved by consensus and discussion with a third reviewer.

### 2.5. Effect size estimation

The Review Manager V.5.3 software was used for the meta-analysis. The I^2^ and Cochran-Q tests were used to assess the heterogeneity between studies. Statistical significance was set at a *P*-value of < 0.05. When the *P*-value is >0.1 or I^2^ is < 50%, the fixed-effect model was used, and when *P*-value of < 0.1 or I^2^ is ≥50%, the random-effect model was used. The mean difference (MD) or the standardized mean difference (SMD), as well as the 95% confidence interval (CI), were computed for continuous data. When the quantitative evaluation was unavailable, we provided a qualitative description of the individual study results. Publication bias was assessed using funnel plots.

### 2.6. Quantitative data synthesis and analysis

For quantitative data synthesis, the estimated combined effect was calculated by comparing the changes between the intervention and control groups at the end of the study period. We assessed the effect of stimulation on the patients' symptoms and compared it with sham samples, using the total score of each scale.

## 3. Results

### 3.1. Study characteristics

A total of 1,625 relevant studies were obtained from the seven electronic databases. Eight studies involving 272 participants were selected and published between 2014 and 2022; three studies were in Chinese, and five were in English. The detailed screening process is shown in Supplemental Figure 1. The meta-registration number is INPLASY2022100025, and the DOI is 10.37766/inplasy2022.10.0025.

### 3.2. Risk of bias and methodological quality of included studies

The details of the included studies and the quality assessment results are shown in Tables 1, 2, respectively. In five studies, a computer or a random table was used to generate random sequences, and three studies only mentioned random allocation without describing it in detail. Two studies described allocation concealment in detail. The most common source of methodological bias was the lack of double blinding. Only three of the eight included studies reported the blinding of the assessors.

**Table 1:** Characteristics of included studies.

**Referenes**	**Age sample (years)**	**N(E/C)**	**Intervention (E/C)**	**Diagnosis**	**Stimulation conditions**	**Target Location**	**Outcomes**
Chen et al. (17)	37.78 ± 9.28	9/9	TMS/ sham	SCA3	1 Hz 900 pulses 30 min, 2 weeks	4 cm to the right of the inion 4 cm to the left of the inion	ICARS
Manor et al. (18)	53 ± 9	10/10	TMS/ sham	SCAs	0.2 Hz 100%RMT, 14 cm circular coil, 20 (sessions) 4 (weeks)	4 cm lateral to the right of the inion 4 cm lateral to the left of the inion,	SARA, Gait, TUG
Fei-fei et al. (19)	33.8 ± 7.2	12/12	TMS/ sham	SCAs	5/10 Hz 100% RMT Double coil P/N3110-00, Rapid2 rTMS 20 (sessions),4 weeks	4 cm lateral to the right of the inion, 4 cm lateral to the left of the inion	SARA
Sikandar et al. (20)	20-80	22/22	TMS/ sham	SCA3	1Hz 100% RMT 1800 pulses 14 cm circular coil 15 minutes,2 weeks	4 cm right of the inion, 4 cm lateral to the left of the inion	ICARS, SARA, BBS
Qian et al. (21)	40.64 ± 10.09	11/9	TMS/ sham	SCAs	5Hz 100%RMT 1800 pulses Round coil 3T 18min,2 weeks	4 cm lateral to the right of the inion, 4 cm lateral to the left of the inion	SARA, BBS,
Kim et al. (22)	67.4 ± 7.8	22/10	TMS/ sham	After stroke CA	1Hz 100% RMT 900 pulses 75 mm-diameter 8 coil 15 min, 10 days	2 cm lateral to the midline on the cerebellar hemisphere ipsilateral to the ataxic side	BBS
Tan et al. (23)	57.15 ± 6.87	42/42	Frenkel+TMS/TMS	After stroke CA	1Hz 80% RMT 1200 pulses 20 min,4 weeks	The contralateral primary motor cortex 2 cm before C3 or C4 point in the contralateral cerebral cortex	ICARS, BBS
Cha et al. (24)	61.60 ± 7.76	15/15	TMS+MT/ sham+MT	After stroke CA	1HZ100%RMT900pulses 70 mm-diameter 8 coil 15 min,4 weeks	2 cm below the inion 2 cm latera l to the midline on the cerebellar hemisphere ipsilateral to the ataxic side	TUG

E, experimental group; C, control group; TUG, Timed Up-and-Go Test; ICARS, International Cooperative Ataxia Rating Scale; SARA, Scale for the Assessment and Rating of Ataxia; BBS, Berg Balance Scale; SCA, Spinocerebellar ataxia; CA, cerebellar ataxia; Frenkel, Frenkel gymnastics training; TMS, Transcranial magnetic stimulation; MT, mirror therapy; RMT, resting motor threshold.

**Table 2:** Quality appraisal of the selected articles.

**References**	**Randomization**	**Concealment** **of allocation**	**Double blinding**	**Withdrawals and** **dropouts**	**Jadad score**
Cha et al. (24)	2	2	2	0	6
Chen et al. (17)	1	1	1	1	4
Manor et al. (18)	1	2	1	0	4
Wei et al. (19)	1	1	0	1	3
Sikandar et al. (20)	1	1	1	1	4
Qian et al. (21)	1	1	1	0	3
Kim et al. (22)	2	2	2	1	7
Tan et al. (23)	1	1	1	0	3

### 3.3. Effects of TMS on patients with cerebellar ataxia

#### 3.3.1. The scale for the assessment and rating of ataxia

**Figure 1:**
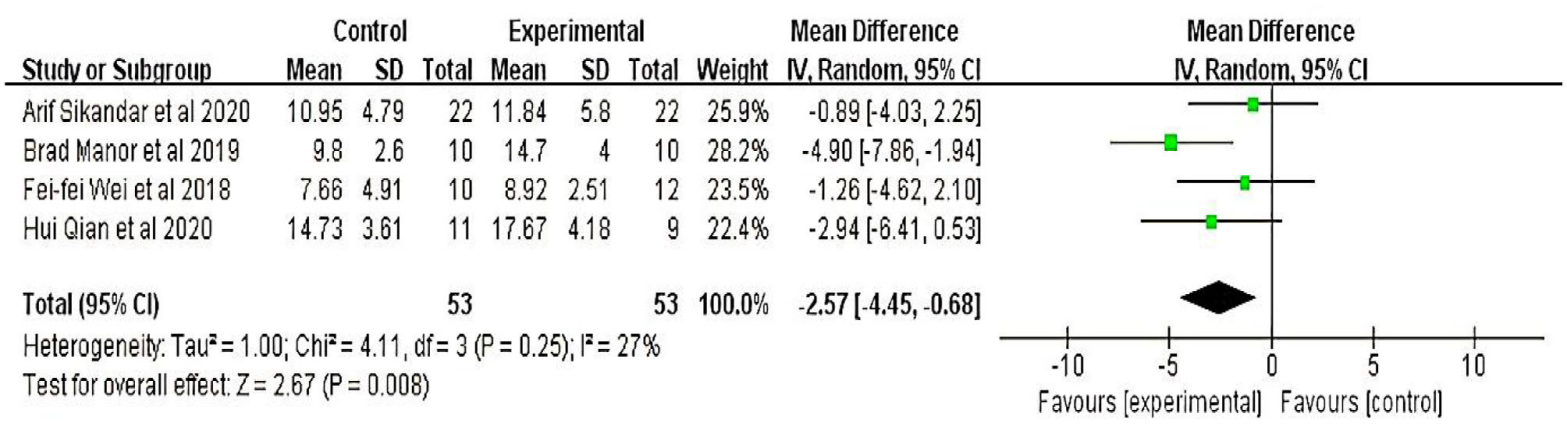
Scale for the assessment and rating of ataxia (SARA).

Four studies (18–21) involving 106 participants evaluated the scale for the Assessment and Rating of Ataxia. Meta-analyses showed that the therapeutic effect of TMS on patients with cerebellar ataxia was significant (MD = −2.60, 95% CI = −0.99 to −4.12; *P* = 0.002) with minor heterogeneity (I^2^ = 27%) (fixed-effect model) (Figure 1).

#### 3.3.2. The international cooperative ataxia rating scale

**Figure 2:**
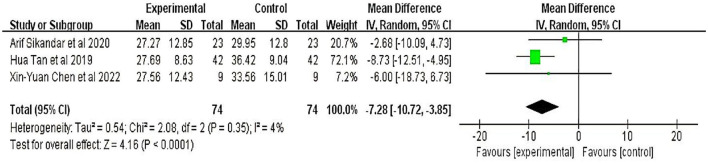
International Cooperative Ataxia Rating Scale (ICARS).

Three studies (17, 20, 23), involving 148 participants, evaluated limb ataxia using the International Cooperative Ataxia Rating Scale. Meta-analyses showed that the therapeutic effect of TMS on patients with cerebellar ataxia was significant (MD = −7.38, 95% CI = −10.64 to −4.13; *P* < 0.01) with minor heterogeneity (I^2^ = 4%) (fixed-effect model) (Figure 2).

#### 3.3.3. The berg balance scale

**Figure 3:**
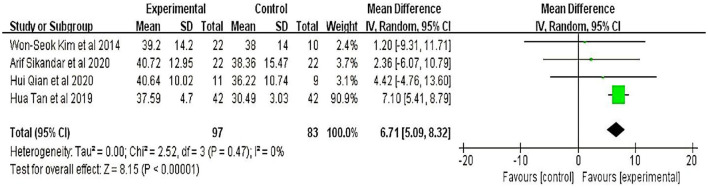
Berg Balance Scale (BBS).

Four studies (20–23) involving 180 participants were evaluated using the Berg Balance Scale. Meta-analyses showed that the therapeutic effect of TMS on patients with cerebellar ataxia was significant (MD = 6.71, 95% CI = 5.09 to 8.32; *P* < 0.01) and without heterogeneity (I^2^ = 0%) (fixed-effect model) (Figure 3).

#### 3.3.4 The timed up-and-go test

**Figure 4:**
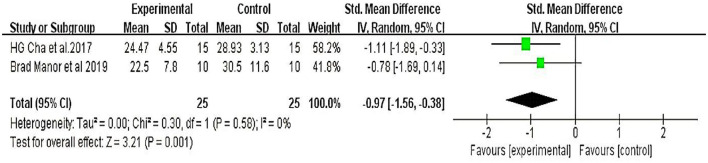
Timed up-and-go test (TUG).

Two studies (18, 24) involving +50 participants evaluated functional mobility with the TUG test. Meta-analyses showed that the therapeutic effect of TMS on patients with cerebellar ataxia was significant (MD = −4.79, 95% CI = −7.45 to −2.13; *P* < 0.01) and without heterogeneity (I^2^ = 0%) (fixed-effect model) (Figure 4).

#### 3.3.5. Publication bias

Funnel plot analysis was not performed due to the limited number of studies.

## 4. Discussion

In this study, we conducted a systematic review and meta-analysis of eight studies involving 272 participants with cerebellar ataxia to evaluate and change the efficacy and safety of TMS applied to cerebellar ataxia and the indicators involved in the ICARS, SARA, BBS, and TUG test. Although TMS has been studied for over 30 years (25), only a few studies have applied it to cerebellar ataxia. According to a literature review published in 2018 (1), TMS could be used as a diagnostic tool in early corticospinal malformation (hereditary ataxia). In a previous review, we discussed the impact of non-invasive brain stimulation on patients with cerebellar ataxia, including tDCS, TMS, and TBS, or as a complementary tool for drug treatment. Our study aimed to determine the TMS efficacy in patients with ataxia, focusing on the effects of TMS in patients with cerebellar disorders of different etiologies, ataxia symptoms, balance changes, and the TMS parameters.

Four studies with participants with spinocerebellar ataxia (SCA) used the Ataxia Assessment and Rating Scale (SARA) for evaluation of the effects of TMS on patients with cerebellar ataxia; the results showed a significant (*P* = 0.002) decrease in the MD between the treatment and control groups. Brad et al. found that TMS may enhance standing balance by improving the capacity to control the speed and magnitude of the postural sway and that such improvements may persist for at least 1 month; the total SARA score decreased more from baseline to 1-month follow-up (F = 9.3, *p* = 0.008, Cohen's d = 1.3, η^2^ = 0.38), suggesting that the long-term effects of TMS in patients with SCA might be more significant. In addition, three studies with TMS intervention frequencies of 10, 5, or 1 Hz had positive results without significant differences. These results suggest that low and high frequencies significantly affect cerebellar ataxia.

Three studies that used ICARS were selected for data synthesis, and all of them showed positive effects and positive results. In two of these three studies, the treatment groups used TMS alone as an intervention, while the patients in one study used TMS + Frenkel gymnastics training. Each study's effect value (ataxia symptoms) suggested that the impact of the TMS treatment alone was the same as that of the TMS combined with rehabilitation training.

**Figure 5:**
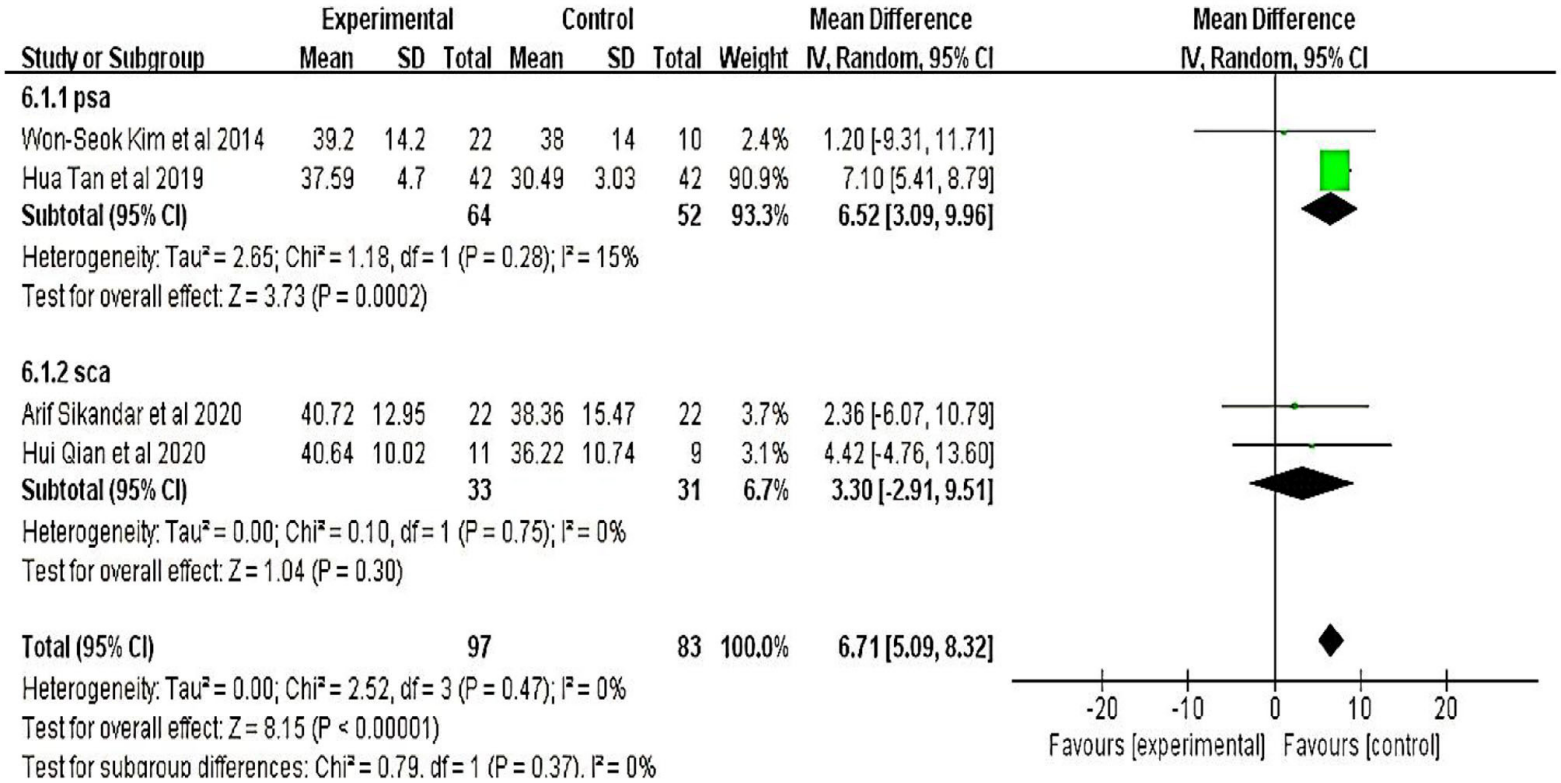
Subgroup analyses of BBS.

Six studies evaluated the improved balance in patients with cerebellar ataxia; four of these studies used the BBS and two used the TUG to assess the function of the ratio. The results showed that the degree of balance in the treatment group improved significantly compared to the control group (*P* < 0.01). Moreover, the cerebellar ataxia etiologies were SCA and post-stroke cerebellar ataxia. We further performed a subgroup analysis and found that the etiological factors of cerebellar ataxia significantly affected the main results (Figure 5), suggesting that the effect of TMS was affected, to some extent, by etiological differences.

The intervention time of four studies was 4 weeks, and the etiology of two of them was SCA. According to the experimental results, their standing posture control was improved in addition to the decline in ataxia scores. The other disease was post-stroke ataxia because TMS combined with other types of training, independent improvement in gait, and functional flexibility were the main outcome measures. There were four studies with durations ranging from 10 to 14 days, and in three of them, the balance was primarily improved. At the time of evaluation, the duration of the intervention may influence changes in the outcome measures.

This review has several limitations; many of the studies included in our review had methodological flaws and small sample sizes. Supplemental Figure 1 and Table 2 show that the most common methodological deficiencies were participants, therapists, and assessors who were blinded to the personal data. Second, the heterogeneity of cerebellar ataxia may confound the results, and each disease may require a specific treatment approach. TMS pulses ranged from 900 to 1,800. The stimulation duration varied from 10 to 4 weeks. This diversity in stimulus patterns may have affected the outcome indicators.

Therefore, large-scale cohort studies and extensive data analysis are still needed to clarify the optimal TMS regimen for treating limb ataxia symptoms. Future TMS studies must be rigorously designed in terms of a randomized parallel group design, an adequate sample size, accurate targeting, and an optimal intervention time window to ensure the quality of the evidence.

## Data availability statement

The original contributions presented in the study are included in the article/Supplementary material, further inquiries can be directed to the corresponding author.

## Author contributions

YW and DZ performed the manuscript writing. LL and JM revised the manuscript for important content. DZ and JW participated in the final design of the study. SJ conceived and designed the research and handled funding and supervision. All authors read and approved the final manuscript.
