# Supplementary figures and images for: Effects of transcranial magnetic stimulation on cerebellar ataxia: A systematic review and meta-analysis
Source: Front Neurol. 2023 Jan 27;14:1049813. doi: 10.3389/fneur.2023.1049813 (PMC9911422; doi:10.3389/fneur.2023.1049813)

**Supplemental Figure 1. Screening process**

**
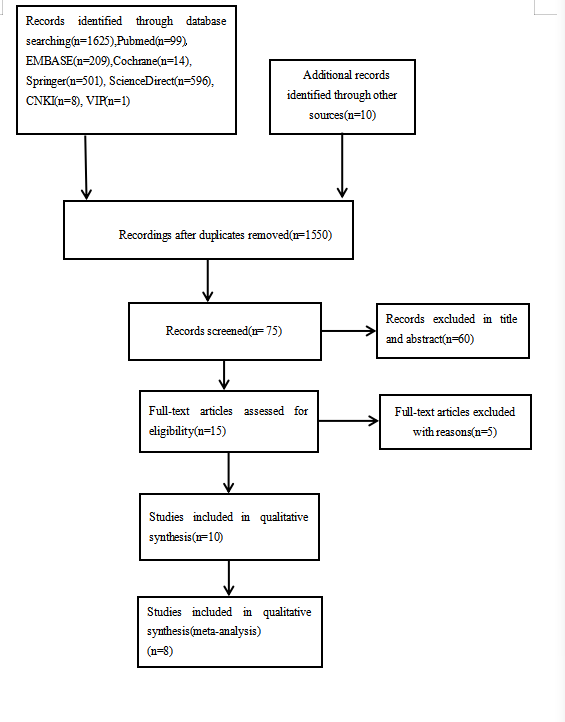
**

**Supplemental Figure 2**


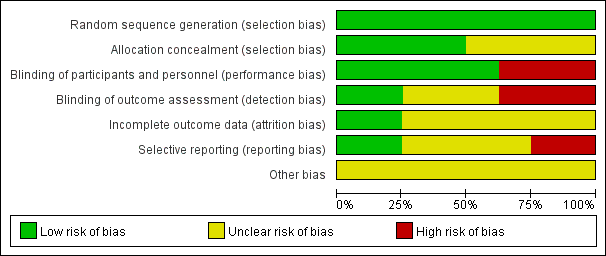


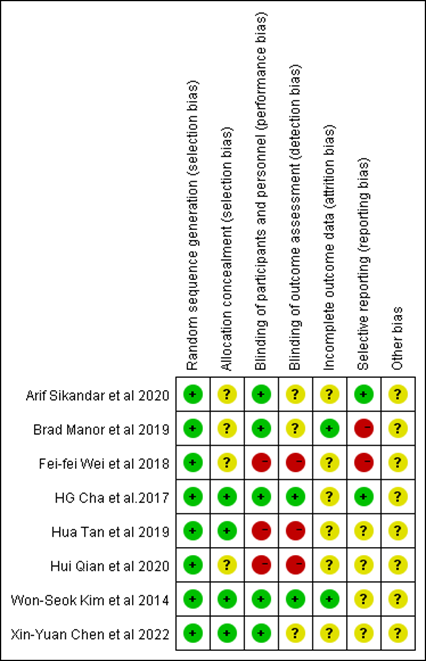

Supplement: Supplementary file 1 [file Data_Sheet_1.docx]
